# Biomechanical analysis of additional vancomycin in articulating knee spacers: determining the threshold for structural failure

**DOI:** 10.1007/s00402-025-06131-0

**Published:** 2025-11-18

**Authors:** Vincent Lallinger, Jan Lang, Benjamin Schloßmacher, Anja Göggelmann, Rainer Burgkart, Rüdiger von Eisenhart-Rothe, Igor Lazic

**Affiliations:** 1 Rechts der Isar Hospital, Munich, Germany; 2 Technical University of Munich, Munich, Germany

**Keywords:** Spacer, PMMA, PJI, Antibiotics, Revision arthroplasty

## Abstract

**Introduction:**

Periprosthetic joint infections (PJI) pose considerable challenges in arthroplasty, with two-stage revisions involving the use of antibiotic-loaded spacers being the prevailing treatment modality for chronic low-grade PJI. Whilst the incorporation of antibiotics has been demonstrated to enhance infection management, the biomechanical impact of such agents on polymethylmethacrylate (PMMA) spacers remains to be elucidated. The present study evaluates the load-bearing capacity of spacers with varying antibiotic concentrations of vancomycin in order to determine structural failure thresholds.

**Materials and methods:**

A total of twenty PMMA knee spacers were subjected to testing, with the samples divided into two distinct groups based on the antibiotic concentration: a low concentration group (5% vancomycin) and a high concentration group (20% vancomycin). The spacers were subjected to uniaxial loading in two configurations: a standard weight-bearing position and a dislocated position with 10° femoral angulation. The breaking forces were measured using a servo-hydraulic testing machine. Statistical analyses were performed using Mann-Whitney-U tests, with significance at *p* < 0.05.

**Results:**

In the standard position, the mean breaking force for the low and high antibiotic groups was 38.7 ± 9.3 kN and 35.5 ± 5.8 kN, respectively (*p* = 0.421). In the dislocated position, breaking forces were significantly lower at 2.2 ± 0.7 kN and 2.0 ± 0.3 kN, respectively (*p* = 0.311). Spacer fractures occurred exclusively in femoral components, with a 17-fold reduction in load capacity in dislocated configurations. The antibiotic concentration of vancomycin exerted no significant effect on biomechanical integrity.

**Conclusion:**

In the experimental ex vivo study, it was demonstrated that vancomycin concentrations of up to 20% by volume in PMMA knee spacers do not significantly affect the structural integrity. However, the positioning of the spacer has been shown to have a significant impact on biomechanical stability, with dislocation having a substantial effect on load capacity.

## Introduction

Periprosthetic joint infections (PJI) are a significant medical and socioeconomic challenge in arthroplasty [1–4]. A range of therapeutic approaches exists with modifications depending on the time and severity of the infection [5].

The two-staged revision, which involves the removal of the infected implant and temporary implantation of an antibiotic-loaded bone cement (ALBC) spacer followed by subsequent definitive prosthesis implantation, has been established as widely accepted standard treatment for chronic low-grade PJI [5–9].

In addition to the function of maintaining adequate volume for the subsequent implant, the utilisation of spacers has been hypothesised to increase local antibiotic concentration [10, 11]. As demonstrated in the extant literature, local antibiotic therapy using antibiotic-loaded bone cement spacers has been shown to improve outcomes in cases of (PJI) [10, 12]. In a meta-analysis, Luu et al. demonstrated that the utilisation of antibiotic-loaded bone cement spacers resulted in a 7% absolute risk reduction in infection recurrence [13]. Meanwhile, studies by Stockley et al. and Hart et al. reported infection eradication rates ranging from 87.7% to 89% [12, 14–16].

However, the precise threshold at which the addition of vancomycin to spacers compromises their biomechanical strength remains to be elucidated. The variability amongst manufacturers of bone cement, in conjunction with the divergent methodologies employed for the incorporation of antibiotics manually, further serves to impede the comparability of findings across studies. A number of studies have indicated that the mechanical strength of bone cement is decreased by increasing concentrations of antibiotic. This effect has been observed with particular clarity in the context of prolonged incorporation and exposure to simulated body fluid washout conditions [17, 18]. The extant literature pertaining to the biomechanical properties of polymethylmethacrylate (PMMA) principally refers to studies on the fixation of cemented total hip arthroplasties [8, 19–21]. In light of the findings, Lautenschlager et al. advanced the “10% rule,” which stipulates that the addition of antibiotics to PMMA should not exceed 10% of the PMMA volume in order to ensure the preservation of mechanical strength [17, 19]. However, the mechanical loading tests supporting this rule were predominantly employing conventional biomechanical testing protocols, such as ISO-16,402, involving unilateral or cyclic loading of PMMA pellets.

Consequently, the design of this test method and its resulting thresholds cannot be fully applied to temporary spacers in periprosthetic joint infection (PJI), as these more closely resemble actual arthroplasty implants. In particular, disparities in geometry, contact surfaces, and their positioning relative to the bone are pivotal factors influencing spacer stability. These factors have been shown to have a significant impact on the risk of breakage, dislocation, and overall clinical outcomes. Such complications may necessitate surgical revision, which carries with it a significant degree of risk to patients. Consequently, a thorough understanding of the biomechanical properties of vancomycin-loaded bone cement spacers is essential. Nevertheless, extant knowledge on this topic remains limited.

The objective of this study was to evaluate the biomechanical load-bearing capacity of spacers in relation to their antibiotic content.

## Materials and methods

A total of 20 articulating PMMA spacers, consisting of independent femoral and tibial components, were moulded according to the manufacturer’s instructions (Heraeus Medical Copal knee moulds, Size M, Tibial Height 12 mm; utilising 80 g PALACOS R each femoral and tibial). In the experiment, 10 spacers were mixed with 2 g of vancomycin powder per 40 g batch PMMA (5% by volume – group Low) or 8 g of vancomycin powder per 40 g batch PMMA (20% by volume – group High). PMMA mixing was performed using a vacuum system (Heraeus Medical PALAMIX), in accordance with the manufacturer’s instructions. This method is also the standard procedure applied in our clinical practice.

Subsequently, the spacers were immersed in phosphate-buffered saline (PBS) for a duration of 24 h. This was done in order to simulate the initial elution of the antibiotic.

**Fig. 1:**
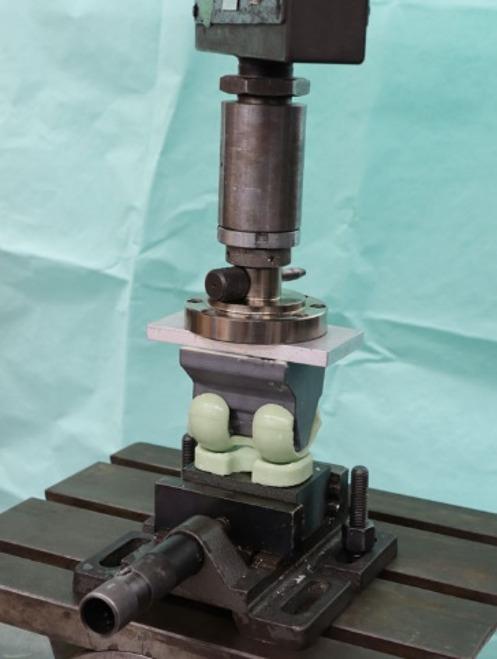
Biomechanical test setup. The antibiotic-loaded spacer is mounted within the testing apparatus. A custom 3D-printed fixture was used to ensure optimal load transfer at the femoral component. The axial load is applied from above

**Fig. 2:**
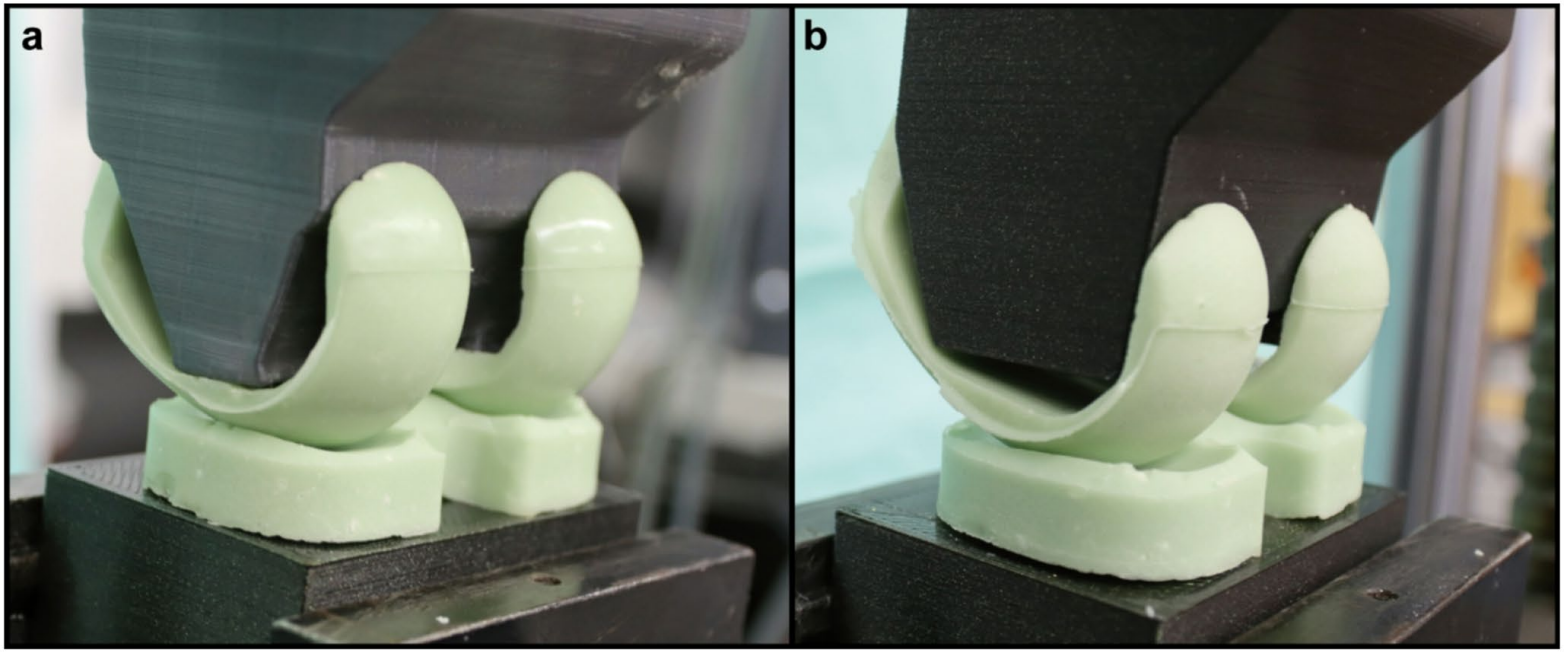
Knee spacers in different femoral mounting configurations. **a** Femoral mount designed to match the distal contact surface of the femoral spacer component, simulating weight-bearing in an extended knee joint (“standard position” test). Owing to the planar support provided by the 3D-printed fixture, an even load distribution is achieved. **b** Femoral mount positioned to create a 10° angulation in the sagittal plane of the femoral spacer component, simulating a dislocated femoral component (“dislocation” test). This angulation results in a tilted positioning and an uneven load distribution, producing peak stresses in the condylar regions

Each spacer was mounted in the biomechanical test device using a 3D-printed frame that simulates femoral and tibial contact surfaces. The spacer is intraoperatively and mechanically held in position by the 3D-printed frame (material: polylactic acid (PLA); 3D printer: UltiMaker 2+, Ultimaker B.V., Geldermalsen, Netherlands). The frame itself was fixed orthogonally in a servo-hydraulic testing machine (Amsler HC 10, Zwick/Roell, Ulm, Germany) (see Fig. 1). Uniaxial loading was applied at a displacement rate of 10 mm per minute and increased continuously until fracture of the femoral or tibial spacer component occurred, or until the maximum load capacity of the testing machine (50kN) was reached. The respective breaking load was recorded. Two distinct experiments were conducted, employing an equal number of spacers in each group: In the initial trial, a femoral mount that corresponded to the distal contact surface of the femoral spacer component was employed, with the objective of simulating weight bearing in an extended knee joint (see Fig. 2a, designated as the ‘standard position’). In the second test, a femoral mount was applied to create a 10° angulation in the sagittal plane of the femoral spacer component. This was done to simulate femoral component dislocation in 10° extension (see Fig. 2b, ‘dislocation’). The experimental setup was designed to replicate the clinical situation as closely as possible and was developed in collaboration with the engineers at our institute. The applied loading protocol was intended to simulate the progressive weight-bearing that occurs in patients during standing, both with the knee in full extension and flexion.

The analysis of the data was conducted utilising SPSS Statistics software, version 29.0, developed by IBM and based in New York, USA. The normality of the distribution was tested using the Kolmogorov-Smirnov test, and the continuous variables were expressed as the mean ± standard deviation. The statistical analysis was.

The Mann-Whitney U-test was the foundation for this study. The statistical significance was set at a value of *p* < 0.05.

## Results

In the ‘standard position’ test, the mean force until breakage in the low and high groups was 38.7kN ± 9.3kN and 35.5kN ± 5.8kN, respectively (*p* = 0.421). In the ‘dislocation’-test, the mean force until breakage in the low and high group was 2.2kN ± 0.7kN vs. 2.0kN ± 0.3kN, respectively (*p* = 0.311).

**Fig. 3:**
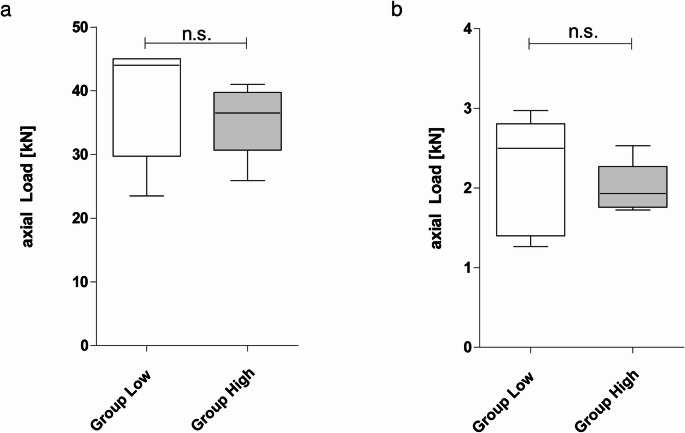
Boxplot showing the mean failure force of the spacers under standard position loading (**a**) and dislocation loading conditions (**b**). The Y-axis indicates the applied axial load (kN), and the X-axis denotes the two groups compared: Low-dose and High-dose vancomycin

A significant discrepancy was observed between the standard position and dislocation test groups. In Group Low, the force required to achieve breakage was smaller by a factor of 17.6. In Group High, the force required to achieve breakage was smaller by a factor of 17.8. The results of the study are summarised in Table 1; Fig. 3.

The fractures occurred exclusively on the femoral components in the area of the condyles, with no fracture of the tibial components being recorded.

**Table 1:** Mean force until breakage of spacers in standard position test and dislocation test

	Group Low	Group High	
Standard position	38.7kN ± 9.3kN	35.5kN ± 5.8kN	*P* = 0.421
Dislocation	2.2kN ± 0.7kN	2.0kN ± 0.3kN	*P* = 0.311
	*P* < 0.001	P < = 0.001	

## Discussion

The present study investigated the biomechanical implications of varying vancomycin concentrations in knee spacers. The most significant finding is that no clinically significant biomechanical differences have been identified in relation to varying antibiotic concentrations. In the context of cemented total hip arthroplasty (THA), it was hypothesised that polymethylmethacrylate (PMMA), the material used to connect the bone to the implant, would become more porous due to the addition of antibiotics. This, in turn, would reduce the mechanical stability of the implant [22]. A critical value of 10% of the total PMMA content was identified [23]. In consideration of the findings of this study, it can be concluded that the aforementioned hypothesis is invalid in the context of knee spacers. Despite the incorporation of 20% vancomycin into the PMMA, no substantial deterioration in mechanical integrity was observed in this study. A substantial corpus of research has been dedicated to the mechanical properties of PMMA; nevertheless, the majority of this research has been focused on conventional compression and elongation testing of PMMA blocks or pellets. However, these studies do not take into account the implications or potential clinical complications associated with the use of molded knee spacers in the context of PJI. It is evident that the extant literature pertaining to the biomechanical properties of knee spacers is somewhat limited [24–30]. Chong et al. applied forces of up to 5000 N to the same prefabricated spacers that were utilised in the experimental setup. However, no fractured spacers were observed during the experiment. The objective of the study was to compare the mechanical stability of the prefabricated spacers with that of hand-moulded spacers. The spacers were loaded with 5% of local antibiotics, specifically 1 g of gentamicin and 1 g of vancomycin in 40 g of polymethyl methacrylate (PMMA) [31].

Egenolf et al. investigated the biomechanical properties of static knee spacers. Their findings revealed no clinically significant alterations with regard to deformation, stiffness, or spacer movement under loads of up to 1000 N [32]. Bitsch et al. conducted a series of in vitro experiments focusing on the analysis of wear characteristics of the same articulating knee spacers investigated in the present study. The findings of the study indicated that the spacers met the requirements of ISO 5833. However, the compressive strength assessments conducted utilised cylindrical specimens, thereby restricting the clinical relevance of the findings with respect to spacer fracture risk. Furthermore, the study did not delve into the potential repercussions of incorporating antibiotics on the mechanical properties of the spacers [33].

In contrast, Kwong et al. reported findings that are not aligned with our results. In their study, the addition of 6 g of vancomycin to 40 g of PMMA resulted in a significant reduction in biomechanical strength compared with the use of 2 g of vancomycin. However, it should be noted that the authors employed different PMMA formulations (gentamicin–clindamycin and gentamicin–vancomycin) than those used in our investigation [34].

Gandomkarzadeh et al. conducted an interesting study and demonstrated a marked decrease in the mechanical strength of PMMA upon the addition of 2.5% (v/v) vancomycin, which is generally in contrast to the findings of this study. However, in their experimental setup, the vancomycin-loaded PMMA was cast into cylindrical and strip-shaped specimens rather than into geometries comparable to those used in our study. In the course of the compression tests conducted, the lowest fracture strength that was measured for samples containing vancomycin was approximately 50 MPa (equivalent to 50 kN/m²). Whilst concurring with the aforementioned interpretation of the mechanical results, it is this author’s opinion that this reduction is not of any clinical relevance, on the grounds that patients with such spacers are unlikely to generate comparable loading forces in vivo [35].

It is interesting to note that, irrespective of the antibiotic concentration, extraordinarily high forces were observed in the standard position, which was designed to simulate the weight-bearing of an extended leg. This is especially evident in relation to the load capacity of bones and the strain that actual arthroplasties are exposed to on a daily basis.

Contemporary total knee arthroplasties are capable of supporting multiple body weights. Kutzner et al. reported in vivo knee joint load measurements which indicated that daily living loads for an 80 kg individual ranged from 220 to 350% of body weight, corresponding to approximately 1700–2700 N [36]. It is reasonable to hypothesise that, under idealised conditions, full weight-bearing on a correctly positioned knee spacer could be mechanically feasible, based on the present ex vivo uniaxial load-to-failure data. However, it is important to exercise caution when interpreting these results, as the experimental setup does not account for factors that are clinically relevant. These include cyclic loading, variations in patient bone quality, and the overall stability of the spacer with respect to the bone construct. These aspects are likely to influence the in vivo performance and must be considered before translating the present findings into clinical recommendations. In contrast, earlier research employing cadaver femora has demonstrated that human femora necessitate fracture loads that are lower than those observed in the spacers examined in the present study. Perez et al. conducted compression tests on seven human femurs, revealing a breaking load ranging from 3,400 to 4,500 N. Notably, fractures were predominantly observed at the proximal end of the femur in six out of the seven specimens, with only a single case exhibiting a fracture at the distal femur [37].

Consequently, it can be hypothesised that fractures to the bone are more probable than spacer breakages under increasing load when the spacer components are correctly positioned. However, it is important to note that the load capacity is significantly reduced for dislocated spacer components. A dislocated femoral component was found to have a significant effect on fracture load, reducing it by a factor of 17. In this case, the daily living loads previously described by Kutzner et al. would be sufficient to break the spacer [36]. Spacer breakages remain a severe complication necessitating surgical intervention. In the analysis conducted by Struelens et al., which encompassed 154 knee spacers, complications were observed in 57% of cases. Of these complications, spacer breakage was reported in seven cases, constituting 5% of the total complications. It is noteworthy that all of the cases of spacer breakage occurred in the femoral region [38]. This finding is consistent with the results of the present study, which suggests a higher likelihood of femoral component fractures, likely due to the thinner thickness of the femoral component in comparison to the tibial component. The dislocation of the femoral component has been demonstrated to pose a significant risk for spacer breakages, thereby emphasising the necessity for meticulous fitting to the host’s bone.

It is noteworthy that even at elevated vancomycin concentrations, no substantial disparities were observed in the load test of dislocated femoral components. Consequently, it can be deduced that the spacer positioning has a greater impact on spacer breakage than the antibiotic concentration.

The manufacturer’s recommendations for knee spacers concerning loading and range of motion are typically contingent on the surgeon’s discretion. The findings of this study lend support to the hypothesis that antibiotic-loaded bone cement spacers may be efficacious in the mitigation of force exerted by full weight bearing. Nevertheless, it must be noted that this assertion cannot be definitively substantiated, as the investigation has omitted a number of crucial elements in this biomechanical analysis. Further studies are required to assess tissue compatibility at higher antibiotic concentrations. In a study by Antoci et al., the local toxicity of ciprofloxacin, tobramycin, and vancomycin was evaluated, and it was observed that there was a reduction in osteoblast andchondrocyte viability with increasing antibiotic concentrations. Vancomycin, a frequently employed antibiotic in arthroplasty, exhibited the least impact on cell viability. Nevertheless, definitive recommendations concerning maximum safe doses or minimum inhibitory concentrations remain to be established. Concerns have already been raised regarding the systemic toxicity of locally applied antibiotics, particularly vancomycin. In a related study, Wahl et al. reported serum vancomycin levels of less than 10 mg/L following local administration of up to 6 g applied to calcium sulphate. The occurrence of nephrotoxicity, as indicated by acute renal insufficiency, was exclusively observed at serum concentrations greater than 10 mg/L [39, 40].

The present study is subject to various limitations. Initially, the loading tests were conducted uniaxially, exclusively on the spacers. However, it is important to note that multidirectional loading tests are possible with articulating spacers. Consequently, it is not possible to draw any conclusions regarding the load distribution through the spacer. In order to provide further recommendations regarding gait, loading tests should be conducted in a flexed knee design. Secondly, the study did not analyse the effect of loading forces on human bones when knee spacers are placed within them. The approach was deliberately chosen on the basis of the literature and the assumption that fractures occur earlier in human bones. Thirdly, it is important to note that the spacers were not subjected to simulated in vivo conditions, such as resting periods in synovial-like fluid. As has been previously documented, the decline in the compression strength of PMMA following incubation has been observed [17, 18, 41, 42]. Bitsch et al. demonstrated that the mechanical properties of the spacer cement used in this study are comparable to those of bone cements after a 50-day storage period at 37 °C in water, in accordance with ISO 5833 standard values. However, their analysis was conducted on PMMA blocks rather than molded spacers, limiting its direct applicability to clinical scenarios. Consequently, further biomechanical evaluation of knee spacers within this context is necessary.

Fourthly, it should be noted that no cyclic loading tests were carried out. This mission is regarded as secondary, given that spacers are only implanted temporarily and are not intended for prolonged implantation.

Furthermore, the investigation was conducted on a single subtype of articulating spacer. Consequently, it is not possible for this study to provide a recommendation that is applicable in all cases.

This study presents an experimental setup developed in collaboration between orthopaedic surgeons and engineers to closely replicate the clinical conditions of articulating knee spacers. Notwithstanding the technical limitation of the testing machine’s maximum load capacity (50 kN), the applied loads remained within a clinically relevant range. Further research utilising higher load capacities may offer further insights into potential discrepancies between antibiotic dosages.

The findings contribute to a more evidence-based understanding of spacer loading and provide practical insights for clinical decision-making. However, it is imperative to exercise caution when interpreting the results, given the restricted generalizability to alternative commercial or hand-moulded spacer systems.

In summary, within the experimental setting, the incorporation of vancomycin into articulating knee spacers at concentrations of up to 20% by volume does not have a detrimental effect on their biomechanical integrity. It is imperative to reiterate that this study was conducted utilising an ex vivo experimental setup. However, it is imperative to emphasise the necessity of meticulous attention to the precise positioning of the spacer components, irrespective of the antibiotic concentration. Further studies are required to investigate the potential local or systemic side effects of higher antibiotic concentrations in knee spacers.

At this juncture, it is imperative to underscore that this study was conducted exclusively through in vitro experiments, necessitating further research to validate the findings. Furthermore, the specially manufactured spacers have not been subject to the approval process of the CE and FDA. In accordance with German medical device regulations, the addition of antibiotics to bone cement is classified as an intraoperative modification, for which the operating surgeon assumes full responsibility.

## Data Availability

No datasets were generated or analysed during the current study.
